# PredT4SE-Stack: Prediction of Bacterial Type IV Secreted Effectors From Protein Sequences Using a Stacked Ensemble Method

**DOI:** 10.3389/fmicb.2018.02571

**Published:** 2018-10-26

**Authors:** Yi Xiong, Qiankun Wang, Junchen Yang, Xiaolei Zhu, Dong-Qing Wei

**Affiliations:** ^1^State Key Laboratory of Microbial Metabolism, School of Life Sciences and Biotechnology, Shanghai Jiao Tong University, Shanghai, China; ^2^School of Sciences, Anhui Agricultural University, Hefei, China

**Keywords:** type IV secreted effector, sequence information, position specific scoring matrix, machine learning, stacked ensemble method

## Abstract

Gram-negative bacteria use various secretion systems to deliver their secreted effectors. Among them, type IV secretion system exists widely in a variety of bacterial species, and secretes type IV secreted effectors (T4SEs), which play vital roles in host-pathogen interactions. However, experimental approaches to identify T4SEs are time- and resource-consuming. In the present study, we aim to develop an *in silico* stacked ensemble method to predict whether a protein is an effector of type IV secretion system or not based on its sequence information. The protein sequences were encoded by the feature of position specific scoring matrix (PSSM)-composition by summing rows that correspond to the same amino acid residues in PSSM profiles. Based on the PSSM-composition features, we develop a stacked ensemble model PredT4SE-Stack to predict T4SEs, which utilized an ensemble of base-classifiers implemented by various machine learning algorithms, such as support vector machine, gradient boosting machine, and extremely randomized trees, to generate outputs for the meta-classifier in the classification system. Our results demonstrated that the framework of PredT4SE-Stack was a feasible and effective way to accurately identify T4SEs based on protein sequence information. The datasets and source code of PredT4SE-Stack are freely available at http://xbioinfo.sjtu.edu.cn/PredT4SE_Stack/index.php.

## Introduction

Gram-negative bacteria use various secretion systems to deliver their secreted substrates (also called as effectors) from the bacterial cytosol into host cells, which can promote virulence and cause diseases. Until now, eight different secretion systems (type I to type VIII) have been found in Gram-negative bacteria, which differ from each other in their outer membrane secretion mechanisms. There are a number of well-organized databases or web resource on collecting experimentally validated effectors of Type III, IV, and VI secretion systems (Bi et al., 2013; Li et al., 2015; Eichinger et al., 2016; An et al., 2017). Among them, type IV secretion system (T4SS) exists widely in a variety of bacterial species, such as *Bordetella pertussis*, *Helicobacter pylori*, *Coxiella burnetii*, and *Legionella pneumophila* (Chandran et al., 2009; Fronzes et al., 2009; Lifshitz et al., 2013). T4SS specifically secretes type IV secreted effectors (T4SEs), which vary widely across bacterial species. T4SEs mimic the function of host proteins, exert vital functions in cytoplasm of infected eukaryotic cells and play crucial roles in host-pathogen interactions. Accurate and reliable identification of T4SEs is a crucial step toward the understanding of the pathogenic mechanism of T4SS. Due to the biological significance of T4SEs, a number of experimental approaches have been developed to identify novel T4SEs such as fusion protein report assays and secretion apparatus. However, these experimental approaches are time- and resource-consuming. It is highly desirable to develop *in silico* classification models to accurately predict type IV secreted effectors of T4SS based on protein sequence information.

In the last decade, several computational approaches using machine learning (ML) algorithms were developed to predict T4SEs based on protein sequence information. A pioneering method proposed by Burstein et al. (2009) formulated the task of identifying T4SEs on Legionella pneumophila genome as a classification problem using various ML algorithms, including naïve Bayes, Bayesian networks, support vector machine (SVM), Neural networks, and a voting algorithm that is based on these four algorithms. The input features of these algorithms include taxonomical dispersion, regulatory data, genomic organization, and similarity to eukaryotic proteomes (Burstein et al., 2009). Later, the same group developed a hidden semi-Markov model (HSMM) to characterize the amino acid composition of the secretion signal for identification of T4SEs across species (Lifshitz et al., 2013). Chen et al. (2010) used the similar ML-based model as the previous study (Burstein et al., 2009) to predict putative T4SEs in Coxiella burnetii genome, which helped narrow the number of potential targets for subsequent experimental validation. T4EffPred is a SVM-based prediction tool for identifying T4SEs based on four types of sequence-derived features, which were calculated from amino acid composition (AAC) and position specific scoring matrix (PSSM) profiles (Zou et al., 2013). T4SEpre (Wang et al., 2014) is another SVM-based tool for predicting T4SEs from C-terminal 100 amino acids of protein sequences by using AAC, position-specific AAC profiles, and predicted structural features such as secondary structure and solvent accessibility. An et al. (2016) constructed an ensemble model by random forest to integrate the output of the individual predictors (i.e., T4EffPred and T4SEpre) to improve predictive performance. Recently, Wang Y. et al. (2017) presented an effective method to predict T4SEs prediction by integrating information from both 50 N-terminal and 100 C-terminal residues of protein sequences. The model was built by SVM based on three types of features, namely AAC, PSSM, and composition, transition and distribution.

Overall, the currently available computational approaches for prediction of T4SEs vary from one another in terms of the utilized features and ML algorithms. Since the numbers of effectors and non-effectors in genomes are heavily unbalanced (the effectors comprise only a small fraction of a genome), it is highly desirable to develop a prediction method with high precision and high specificity. Otherwise, the number of true positives would easily be overwhelmed by the number of false positives, so that such a predictor is impractical to generate reliable candidates for experimental validation. In the present study, we aim to propose a stacked ensemble model, PredT4SE-Stack, to further improve the prediction performance (i.e., higher precision and specificity) for identifying T4SEs from protein sequence information. The stacked generalization approach (Wolpert, 1992) consists of an ensemble of base classifiers whose outputs are further learned by a meta-classifier to model the relationship between the ensemble outputs and the actual classes/labels. To construct the model, the protein sequences are firstly encoded by the feature of PSSM-composition by summing rows that correspond to the same amino acid residues in PSSM profiles. Based on the PSSM-composition features, a total of eight types of ML-based algorithms (including advanced ML techniques) are used to build base-classifiers in the first stage. Then, the optimal combination of base-classifiers is searched, and the output of these selected base-classifiers are utilized as input for a meta-classifier at the second stage. Our experimental results on both cross validation and independent tests demonstrated that the framework of PredT4SE-Stack is a feasible and effective way to accurately identify T4SEs based on protein sequence information. It also has achieved better performance than previously published methods.

## Materials and Methods

### Dataset

In this study, the same benchmark dataset curated by Wang Y. et al. (2017) was used to evaluate the performance of our proposed method. The dataset consists of 1,765 protein sequences across multiple bacterial species, categorized into two classes (380 T4SEs as the positive class and 1,385 non-T4SEs as the negative class). These proteins in this dataset have mutual sequence identity no more than 30%. The 1,765 protein sequences were divided into two subsets for cross validation in the training and the independent testing, respectively. The training dataset (Train-915) are composed of 915 sequences, among which 305 T4SE sequences were randomly selected from positive class, and 610 non-T4SE sequences were randomly selected from negative class. The dataset of Train-915 was further randomly divided into five subsets (or folds) with an equal number of protein sequences for cross validation to attain the optimized model. In each of the five validations, 4 of the 5-folds were used for training and the remaining one for testing, which was repeated for five times. The testing dataset (Test-850) included the remaining 75 T4SE sequences as positive samples and 775 non-T4SE sequences as negative samples for independent testing.

### Feature Representation of Protein Sequence Samples

One of the key problems in designing a predictor based on machine learning is how to encode a protein sequence as an informative feature vector enriched with highly discriminative information. In the present section, we describe how to formulate an effective mathematical expression that describes protein sequences in the training and testing data sets.

**FIGURE 1:**
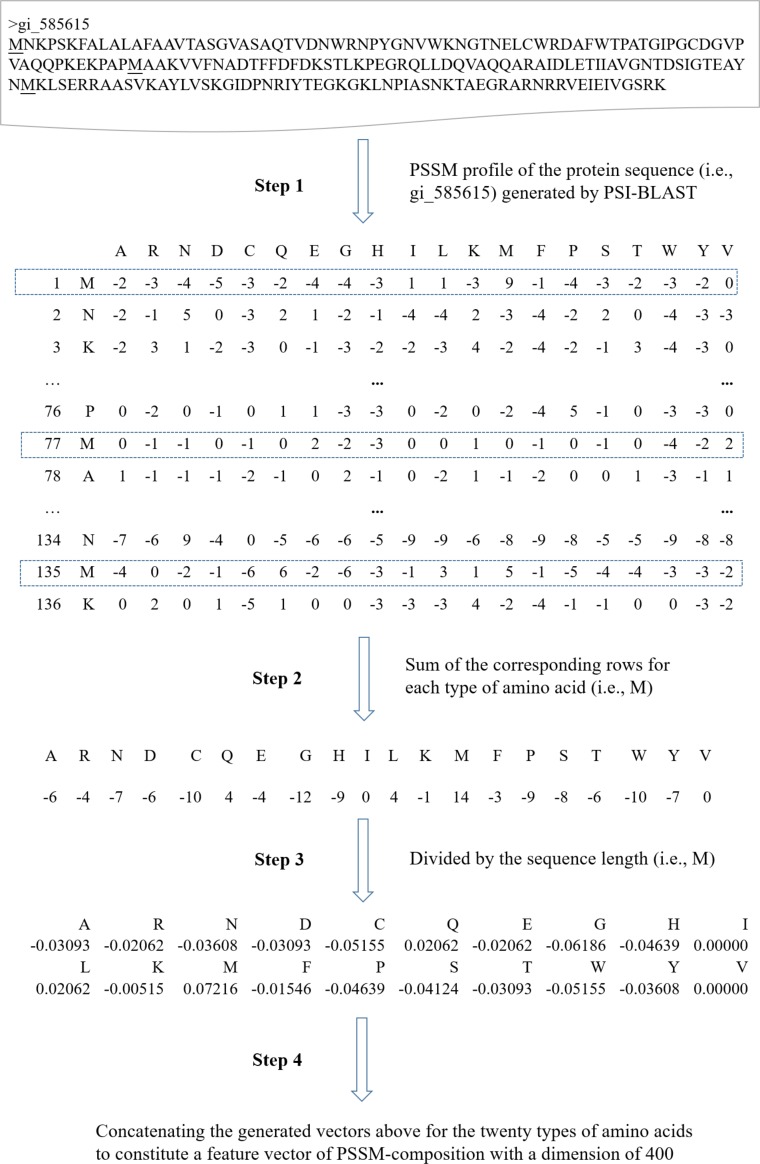
The illustration of PSSM-composition profile calculation for a query sequence.

The protein sequence profile (i.e., PSSM) is a powerful representation of residue or sequence information of proteins. It has achieved good performance on a number of bioinformatics applications such as functional residues prediction and protein function prediction (Xiong et al., 2011a,b, 2012; Zhu et al., 2013; Wei et al., 2017a). In this study, PSSMs were generated by three iterations of PSI-BLAST searches against Uniref50 with the BLOSUM62 substitution matrix. The parameter of e-value was set to 0.001. Because ML-based models can only handle vectors with equal lengths for all protein sequence samples, the PSSM of a protein sequence (amino acid length is *L*) has a dimension of *L*^∗^20, which could not be directly used as the input feature vector for machine learning algorithms. Instead, the original PSSM profile was further used to calculate the feature of PSSM-composition by summing rows that correspond to the same amino acid residues in a PSSM profile, in much the same way as the previous studies (Zou et al., 2013; Wang J. et al’s., 2017). The sum value was divided by the length of the protein sequence for each type of amino acid (there is a total of 20 types). Thus, a vector of size at 400 (=20 × 20) is finally used for representing a protein sequence sample. Figure 1 presents the details about how to generate a feature vector of PSSM-composition for a given protein sequence.

### Classification System

The ensemble learning techniques can be categorized into three main types, which include bagging, boosting, and stacked ensemble. It is demonstrated that the ensemble learning techniques can help improve the prediction performance in various bioinformatics applications (Zhang et al., 2012; Lin et al., 2013, 2014; Zou et al., 2015; Li et al., 2016; Yuan et al., 2016; Wan et al., 2017; Iqbal and Hoque, 2018; Mishra et al., 2018; You et al., 2018). In this section, we introduce the components of the two-stage stacked ensemble scheme, including various classification algorithms used as base-classifiers in the first stage, and the input of the meta-classifier in the second stage.

### Base-Classifier

In order to find the optimal combination of base-classifiers in the first stage and the meta-classifier in the second stage, the following eight different machine learning algorithms were exploited: (i) SVM (Cortes and Vapnik, 1995), (ii) Naïve Bayes (NB), (iii) K Nearest Neighbor (KNN), (iv) Logistic Regression (LR), (v) Random Forest (RF) (Breiman, 2001), (vi) Extremely Randomized Trees (ERT) (Geurts et al., 2006), (vii) Gradient Boosting Machine (GBM) (Friedman, 2001), and (viii) eXtreme Gradient Boosting (XGB). The algorithms such as NB, LR, and GBM were implemented by using h2o package in R software. The algorithms of SVM, KNN, RF, ERT, and XGB are implemented by using e1071, caret, randomForest, extraTrees and xgboost packages in R, respectively. The optimal parameters in these algorithms are determined by a grid search strategy.

### Meta-Classifier

**FIGURE 2:**
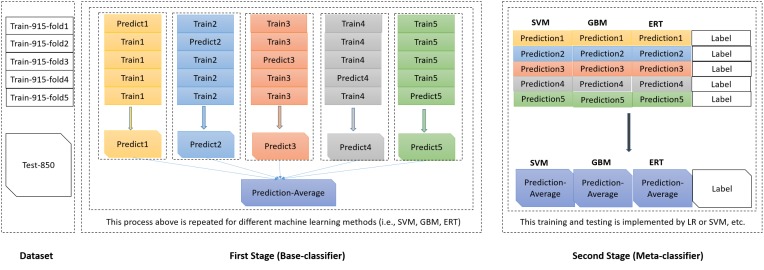
The framework of the stacked ensemble scheme proposed in PredT4SE-Stack.

The meta-classifier in the second level generalization (or stacked generalization) is used to combine the outputs of base-classifiers in an ensemble. In our classification system, we applied a stacked generalization approach proposed by Wolpert (1992), in which an ensemble of base-classifiers are first constructed, whose outputs are used as inputs to a second level of meta-classifier to learn the relationship between the ensemble outputs and the actual classes/labels. The stacked generalization scheme can be viewed as an extension version of cross validation. In the first stage, the base-classifiers were trained with the feature of PSSM-composition of sequences. In the second stage, the prediction class probabilities of the base-classifiers were taken as inputs to the meta-classifier (shown in Figure 2).

### Model Validation Method

To evaluate performances of classification models, the validation methods are mainly consisting of *k*-fold cross validation, leave-one-out cross validation (or called as jackknife test), and independent tests. In *k*-fold cross validation, the sample set is randomly divided into *k* subsets with equal sizes. Of the *k* subsets, only one subset is selected as the validation data for testing the model, and the remaining *k*-1 subsets are used as training data. The cross validation process is then repeated *k* times (the folds), with each of the *k* subsets used exactly once as the validation data. The results from *k* folds are finally averaged. The *k*-fold cross validation method has been widely used as the model validation approach in various bioinformatics applications (Zhu and Mitchell, 2011; Xu et al., 2017; Zeng et al., 2017; Chen X. et al., 2018; He et al., 2018a,d). In the present study, the 5-fold cross validation was used for validation in the training set, and the independent test was used for testing the generalization ability of the proposed method, and comparison with other methods.

### Model Evaluation Metric

In order to assess prediction performances of single-label classification systems, a set of six threshold-dependent metrics are widely used in the bioinformatics studies (Xia et al., 2010; Li et al., 2011; Zhang et al., 2017, 2018a,b,c; He et al., 2018c; Jia et al., 2018; Zhao et al., 2018). They are accuracy (ACC), sensitivity (SE, also called recall), specificity (SP), precision (PR), Matthew’s correlation coefficient (MCC) and F-measure (F_1_). The definitions of these metrics are shown as below.

(1) $$\mathrm{ACC}=\frac{\mathrm{TP}+\mathrm{TN}}{\mathrm{TP}+\mathrm{TN}+\mathrm{FP}+\mathrm{FN}}$$

(2) $$\mathrm{SE}=\frac{\mathrm{TP}}{\mathrm{TP}+\mathrm{FN}}$$

(3) $$\mathrm{SP}=\frac{\mathrm{TN}}{\mathrm{TN}+\mathrm{FP}}$$

(4) $$\mathrm{PR}=\frac{\mathrm{TP}}{\mathrm{TP}+\mathrm{FP}}$$

(5) $$\mathrm{MCC}=\frac{\mathrm{TP}\times \mathrm{TN}-\mathrm{FP}\times \mathrm{FN}}{\sqrt{\left(\mathrm{TP}+\mathrm{FN})\times (\mathrm{TP}+\mathrm{FP})\times (\mathrm{TN}+\mathrm{FP})\times (\mathrm{TN}+\mathrm{FN}\right)}}$$

(6) $$F_{1}=\frac{2\times \mathrm{SE}\times \mathrm{PR}}{\mathrm{SE}+\mathrm{PR}}$$

where TP (true positives) is the number of correctly predicted T4SEs, TN (true negatives) is the number of correctly predicted non-T4SEs, FP (false positives) is the number of non-T4SEs wrongly predicted as T4SEs, and FN (false negatives) is the number of T4SEs wrongly predicted as non-T4SEs.

The receiver operating characteristic (ROC) curve is a plot of the sensitivity versus (1-specificity) for a binary classifier at varying thresholds from 0 to 1 (the threshold is assigned as the probability of the target sequence to be a T4SE in our study). The area under the curve (AUC) can be used as a powerful metric for evaluation performances of classifiers. It is worth mentioning that AUC of ROC (and ACC, MCC) can present overly optimistic assessment of performance of an algorithm on a heavily unbalanced dataset. Therefore, we only used AUC of ROC for evaluation in 5-fold cross validation, but not used it for evaluation in the independent dataset (only 75 proteins are true positives among 850 samples). Instead, the metric of F_1_, which is a harmonic mean of recall (or sensitivity) and precision, is a main metric for evaluating performances of classifiers in the present study.

## Results and Discussion

### Predictive Power of Various Base-Classifiers on Train-915 Dataset

**FIGURE 3:**
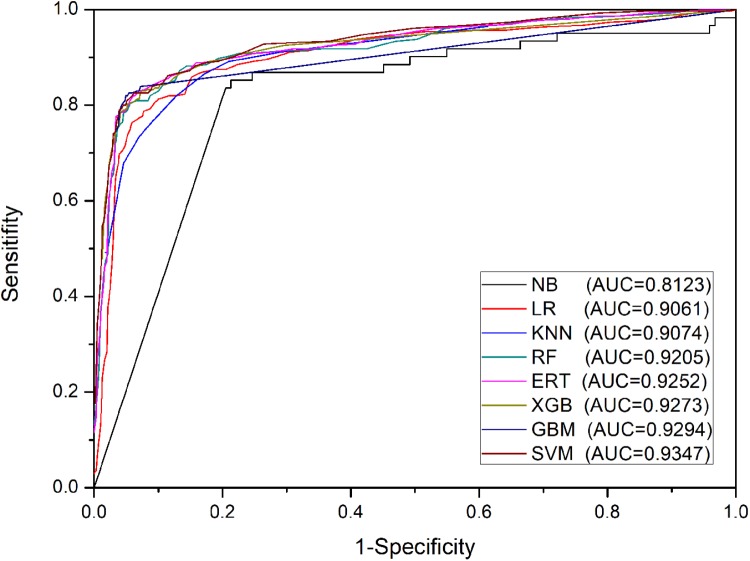
ROC curves of base-classifiers in the first stage on Train-915 dataset using 5-fold cross validation.

The aim of this section is to test the predictive power of base-classifiers based on PSSM-composition profiles for eight different machine learning algorithms on Train-915 dataset using 5-fold cross validation. Experimental results shown in Table 1 indicate that the algorithm of naïve Bayes performed worst on this task. The algorithms of KNN, logistic regression, random forest, and extremely randomized trees performed moderately. The algorithms of support vector machine, extreme gradient boosting, and gradient boosting machine performed best. The results of ROC shown in Figure 3 are mainly in agreement with the findings in Table 1. However, the fact that the AUC-ROC of SVM is higher than that of XGB and GBM indicates that SVM can achieve more stable performance than XGB and GBM using PSSM-composition feature as input in the present task, in regardless of the change of the thresholds. It should be noted that we tried a large number of other types of PSSM-derived features generated by POSSUM tookit (Wang J. et al’s., 2017), and a variety of structural and physiochemical descriptors extracted from protein sequences generated by iFeature tookit (Chen Z. et al., 2018) when we designed the input features of the base-classifiers. Our experimental results demonstrated that the PSSM-composition feature utilized in this study yielded satisfactory performance, which performed better than other types of sequence-based features. Moreover, we attempted to directly combine the PSSM-composition feature with other types of features as the input of the base-classifiers. It was found that the combined features could not significantly produce higher performance than the single type of PSSM-composition feature (data not shown).

**Table 1:** Performance comparison of eight types of base-classifiers in the first stage on Train-915 dataset using 5-fold cross validation.

Method	Parameter	ACC(%)	SE (%)	SP (%)	PR(%)	MCC	F_1_
NB	laplace = 0	73.2	81.0	69.3	57.0	0.476	0.669
KNN	k = 10	85.5	82.0	87.2	76.3	0.680	0.790
LR	family = “binomial”	87.9	74.8	94.4	87.1	0.722	0.803
RF	ntree = 500	88.5	72.5	96.6	91.4	0.738	0.807
ERT	numRandomCuts = 9	89.4	74.8	96.7	92.1	0.759	0.824
SVM	cost = 1, gamma = 2^−8^, kernel = “radial”	90.2	78.0	96.2	91.6	0.777	0.839
XGB	eta = 0.3, max_depth = 6, nrounds = 500, objective = “binary:logistic”	90.1	78.7	95.7	90.4	0.774	0.840
GBM	learn_rate = 0.7, max_depth = 9, ntrees = 50	90.5	80.0	95.7	90.7	0.784	0.847

### Predictive Power of Meta-Classifiers on Train-915 Dataset

Since combining all of the above mentioned base-classifiers in a meta-classifier could not yield optimal prediction performance, it is desirable to search for the optimal combination of base-classifiers. Since RF and ERT are tree-based classifiers, we chose one of them at a time. Because GBM and XGB are boosting-based methods, and XGB is an efficient and scalable implementation of GBM, we chose one of them too. It was found that the combination of SVM, GBM, and ERT achieved the optimal performance, which is in agreement with the finding of study by Pan et al. (2018) on the prediction task of hot spots in protein-RNA interfaces.

**FIGURE 4:**
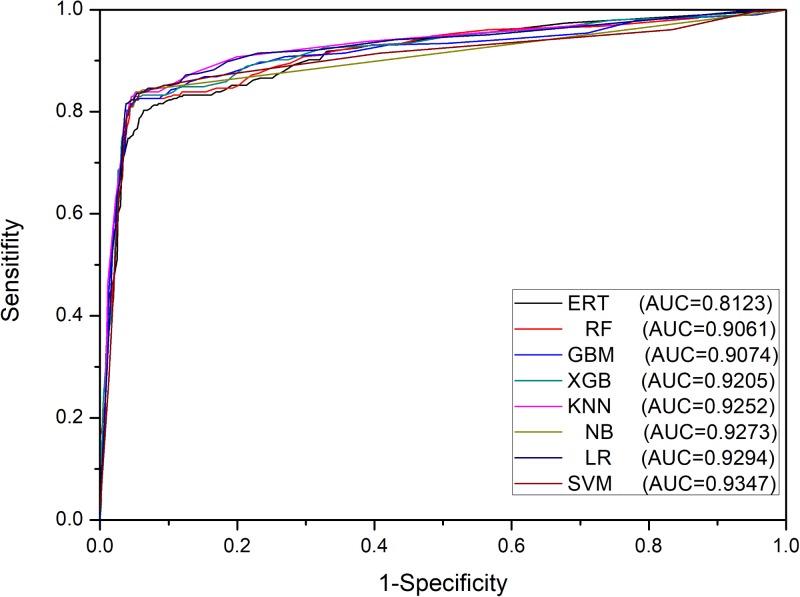
ROC curves of meta-classifiers in the second stage on Train-915 dataset using 5-fold cross validation.

Furthermore, we tested the same set of eight ML methods as the classification algorithms of meta-classifiers to compare their prediction performances. The results in Table 2 showed that all meta-classifiers except the one based on ERT achieved very similar performances, for example, the values of F_1_ are falling in a narrow range from 0.847 to 0.858, whereas the base-classifiers using the same set of ML algorithms are ranging from 0.669 to 0.847 in the first stage. These results can be explained by the fact that the pattern learned from the first stage is effective enough, leading to the similar level of performances at the second stage on the same dataset of Train-915, irrespective of ML algorithms, except ERT (also demonstrated in Figure 4).

**Table 2:** Performance comparison of eight types of meta-classifiers in the second stage on Train-915 dataset using 5-fold cross validation.

Method	Parameter	ACC(%)	SE (%)	SP (%)	PR(%)	MCC	F_1_
ERT	numRandomCuts = 9	88.9	80.3	93.1	86.5	0.752	0.828
RF	ntree = 500	90.4	81.0	95.1	89.7	0.783	0.847
SVM	cost = 10, gamma = 2^−10^, kernel = “radial”	90.6	80.3	95.7	90.7	0.787	0.849
GBM	learn_rate = 0.1, max_depth = 3, ntrees = 50	90.6	82.0	94.9	89.3	0.788	0.851
XGB	eta = 0.1, max_depth = 2, nrounds = 100, objective = “binary:logistic”	90.7	81.3	95.4	90.4	0.791	0.852
NB	laplace = 0	90.9	82.3	95.2	89.9	0.795	0.857
KNN	k = 19	91.0	82.0	95.6	90.5	0.797	0.857
LR	family = “binomial”	91.1	81.0	96.2	91.9	0.800	0.858

### Predictive Power of Meta-Classifiers on Test-850 Dataset

In the section, the prediction performances of meta-classifiers are evaluated on the independent dataset, which is mimicking a true prediction task, since the model trained on one dataset is really tested on an unseen dataset for examining its generalization ability on a new dataset. Table 3 indicated that LR and SVM have top performances on Test-850 dataset. Therefore, both of them can be utilized as the classification algorithms of the meta-classifier in PredT4SE-Stack. Considering the fact that LR is more interpretable than SVM, we could use LR to construct the meta-classifier in our model PredT4SE-Stack. In real application, we will re-train PredT4SE-Stack on a whole dataset consisting of Train-915 and Test-850.

**Table 3:** Performance comparison of eight types of meta-classifiers in the second stage on the independent dataset Test-850.

Method	ACC(%)	SE (%)	SP (%)	PR(%)	MCC	F_1_
XGB	92.4	85.3	93.0	54.2	0.643	0.663
GBM	93.1	88.0	93.5	56.9	0.674	0.691
KNN	93.5	88.0	94.1	58.9	0.688	0.706
RF	93.8	86.7	94.5	60.2	0.691	0.710
NB	93.8	88.0	94.3	60.0	0.696	0.714
ERT	94.0	88.0	94.6	61.1	0.703	0.721
LR	94.4	88.0	95.0	62.9	0.715	0.733
SVM	94.5	86.7	95.2	63.7	0.715	0.734

### Comparison With Previous Studies

The main purpose of this section is to compare our proposed approach PredT4SE-Stack to previously published methods. Performance comparisons among different T4SE prediction approaches are scientifically meaningful only if they train and test their methods on the same dataset. Accordingly, our approach PredT4SE-Stack was only compared with the recently published method proposed by Wang Y. et al. (2017). The first reason is that both two studies used the same benchmark dataset for training and testing. The second reason is that Wang Y. et al. (2017) method had been proved to be improved over other published methods such as T4EffPred (Zou et al., 2013), T4SEpre (Wang et al., 2014), and An et al. (2016) method. Table 4 shows the comparison results between our method with Wang Y. et al. (2017) method. Since the measures of F_1_ and precision are not available in Table 4 in their published study, we firstly calculated the TP, TN, FP, and FN using the sensitivity and specificity of their method, and then calculated F_1_ and precision of Wang Y. et al. (2017) method. The meta-classifier of our PredT4SE-Stack classification system was implemented by SVM or LR. For SVM or LR, the performance (F_1_ = 0.734 or 0.733) of our method is much higher than that (F_1_ = 0.521) of Wang Y. et al. (2017) method. If our SVM-based meta-classifier is tuned on the same recall or sensitivity of 90.7%, our method achieved better performance at specificity, precision, and F_1_, which are 2.4, 4.1, and 4.1% respectively, higher than that of Wang Y. et al. (2017) method. If our LR-based meta-classifier is tuned on the same recall or sensitivity of 90.7%, our method achieved better performance at specificity, precision, and F_1_, which are 3.7, 6.7, and 6.5% respectively, higher than that of Wang Y. et al. (2017) method.

**Table 4:** Performance comparison between our method with the other method on the independent dataset Test-850.

Method	ACC(%)	SE (%)	SP (%)	PR(%)	MCC	F_1_
Wang Y. et al. (2017) method	85.3	90.7	84.8	36.6	0.518	0.521
PredT4SE-Stack (SVM, 0.23)	87.5	90.7	87.2	40.7	0.556	0.562
PredT4SE-Stack (SVM, 0.50)	94.5	86.7	95.2	63.7	0.715	0.734
PredT4SE-Stack (LR, 0.11)	88.7	90.7	88.5	43.3	0.579	0.586
PredT4SE-Stack (LR, 0.50)	94.4	88.0	95.0	62.9	0.715	0.733

## Conclusion

The main goal of the current study is to develop a stacked ensemble model PredT4SE-Stack to predict T4SEs from protein sequence information. The proposed model utilized an ensemble of base-classifiers implemented by SVM, GBM, and ERT to generate outputs for the meta-classifier in the classification system. It was demonstrated that the framework of PredT4SE-Stack was a feasible and effective way to accurately identify T4SEs based on protein sequence information. However, the performance of PredT4SE-Stack can be further improved in several respects. Firstly, the diversity of base-classifiers was implemented by various classification algorithms in the present work. It can be further improved by different features in different base-classifiers. Secondly, inspired by the successful application of feature selection strategies in various bioinformatics tasks (Zou et al., 2016; Wei et al., 2017b, 2018; He et al., 2018b; Manavalan et al., 2018; Qiao et al., 2018; Su et al., 2018; Tang et al., 2018), the predictive power of base-classifiers can be boosted by incorporating an effective feature selection technology on a large pool of sequence-derived features. Moreover, an effective model selection on a large number of candidate base-classifiers will be explored to improve the prediction performance of the meta-classifier. These improvements will be explored in the further study.

## Author Contributions

XZ and D-QW conceived the study. YX and XZ designed the experiments. YX performed the experiments. YX, QW, JY, and XZ analyzed the data. YX and XZ wrote paper. All authors reviewed the manuscript and agreed to this information prior to submission.

## Conflict of Interest Statement

The authors declare that the research was conducted in the absence of any commercial or financial relationships that could be construed as a potential conflict of interest.
